# Ethnic differences in bone geometry between White, Black and South Asian men in the UK^☆^

**DOI:** 10.1016/j.bone.2016.07.018

**Published:** 2016-10

**Authors:** A. Zengin, S.R. Pye, M.J. Cook, J.E. Adams, F.C.W. Wu, T.W. O'Neill, K.A. Ward

**Affiliations:** a Medical Research Council Human Nutrition Research, Cambridge, UK; b Arthritis Research UK Centre for Epidemiology, Centre for Musculoskeletal Research, Institute of Inflammation and Repair, Manchester Academic Health Science Centre, The University of Manchester, Manchester, UK; c Radiology and Manchester Academic Health Science Centre (MAHSC), Manchester Royal Infirmary, Central Manchester University Hospitals NHS Foundation Trust and University of Manchester, Manchester, UK; d Andrology Research Unit, Manchester Academic Health Science Centre (MAHSC), The University of Manchester, Manchester, UK; e NIHR Manchester Musculoskeletal Biomedical Research Unit, Central Manchester NHS Foundation Trust, Manchester Academic Health Science Centre, Manchester, UK; f Department of Rheumatology, Salford Royal NHS Foundation Trust, Salford, UK; g MRC Lifecourse Epidemiology Unit, University of Southampton, Southampton, UK

**Keywords:** Aging, pQCT, Bone, Geometry, Ethnicity, DXA

## Abstract

Relatively little is known about the bone health of ethnic groups within the UK and data are largely restricted to women. The aim of this study was to investigate ethnic differences in areal bone mineral density (aBMD), volumetric bone mineral density (vBMD), bone geometry and strength in UK men.

White European, Black Afro-Caribbean and South Asian men aged over 40 years were recruited from Greater Manchester, UK. aBMD at the spine, hip, femoral neck and whole body were measured by DXA. Bone geometry, strength and vBMD were measured at the radius and tibia using pQCT at the metaphysis (4%) and diaphysis (50% radius; 38% tibia) sites. Adjustments were made for age, weight and height.

Black men had higher aBMD at the whole body, total hip and femoral neck compared to White and South Asian men independent of body size adjustments, with no differences between the latter two groups. White men had longer hip axis lengths than both Black and South Asian men. There were fewer differences in vBMD but White men had significantly lower cortical vBMD at the tibial diaphysis than Black and South Asian men (*p* < 0.001). At the tibia and radius diaphysis, Black men had larger bones with thicker cortices and greater bending strength than the other groups. There were fewer differences between White and South Asian men. At the metaphysis, South Asian men had smaller bones (*p* = 0.02) and lower trabecular vBMD at the tibia (*p* = 0.003). At the diaphysis, after size-correction, South Asian men had similar sized bones but thinner cortices than White men; measures of strength were not broadly reduced in the South Asian men.

Combining pQCT and DXA measurements has given insight into differences in bone phenotype in men from different ethnic backgrounds. Understanding such differences is important in understanding the aetiology of male osteoporosis.

## Highlights

• Ethnic differences in bone health were investigated in UK men using DXA and pQCT
• Areal BMD was highest in Black Afro-Caribbean compared to White and South Asian men
• Black Afro-Caribbean men had the most advantageous geometry and bone distribution
• No differences in areal BMD between South Asian and White men
• At the tibia, South Asians had thinner cortices and lower strength than White men

## 1 Introduction

Osteoporosis is an important health problem through its association with age-related fractures and consequent morbidity and mortality. There are important differences in the occurrence of age related fractures between different regions and populations, which are likely due to variation in bone strength and, or trauma – particularly fall risk. Within the UK there is good evidence about bone health among people of White European background, however, relatively little is known about the underlying bone health in the 10% of people with a non-White European background. Recent results from the UK Clinical Practice Research Datalink report the incidence of hip fracture in White men to be 2.7 times greater than in Black men, and approximately double that of South Asian men [1]. However, there are few data concerning bone mass and strength, and the underlying determinants of fracture risk in UK ethnic minority groups, with no data in men.

One of the first studies in the UK addressing ethnic differences in bone health compared areal BMD (aBMD) in women who were White European, Black Afro-Caribbean and South Asian aged 50–55 years using dual energy X-ray absorptiometry (DXA) [2]. This study showed that lumbar spine and femoral neck aBMD were higher in Black Afro-Caribbean compared to White European women. Conversely South Asian women were reported to have a lower lumbar spine and femoral neck aBMD compared to White European women, however, after correcting for skeletal size the differences at the lumbar spine were attenuated [2]. Similarly, another study showed that in women aged 24–35 years, South Asian women had lower aBMD compared to White European women, however, after adjustments for body or bone size the difference between the groups were attenuated [3]. These studies illustrate the limitations of DXA when describing population differences [4], [5] where body size and habitus differ. Using peripheral quantitative computed tomography (pQCT) has advantages because it measures volumetric BMD (vBMD), cortical and trabecular compartments separately and provides information also about other structural parameters which contribute to bone strength. There are limited data comparing pQCT measurements in different ethnic groups. In the same study reporting no differences in women in size-corrected DXA measurements, the pQCT results showed that South Asian women had lower cortical vBMD, bone mineral content (BMC) and thinner cortices at the radial diaphysis compared to White European women [6]. Despite lower BMC in the South Asian women, bone strength as estimated using the stress strain index (SSI) was similar. Thus, it is possible that bones of pre-menopausal South Asian women may be efficiently adapted to a lower BMC as a result of a different distribution of bone mineral within the periosteal envelope, thereby preserving bone strength [4], [6]. Whether these findings are similar in men remains unknown.

The aim of this study was to investigate ethnic differences in aBMD of the spine, hip and whole body and in vBMD, bone geometry and estimates of bending and torsional bone strength (cross-sectional moment of inertia (CSMI) and stress strain index (SSI)) [7], [8], [9] at the metaphyseal and diaphyseal radius and tibia, using DXA and pQCT in White European, Black Afro-Caribbean and South Asian men living in the UK. We investigated also whether any observed differences could be explained by body weight and height.

## 2 Methods

### 2.1 Participants

Community-dwelling White European men aged 40 years and over were recruited from primary care registers in Manchester (UK) for participation in the European Male Aging Study (EMAS) [10]. Stratified random sampling (by 10 year age band: 40–49 years, 50–59 years, 60–69 years and 70–79 years) was used and subjects were invited by letter of invitation to attend a local clinic for assessment including pQCT and DXA measurements and assessment of height and weight. The men subsequently attended a follow-up assessment of identical measurements a median of 4.3 years later. The results reported here are from the follow-up assessment. During the EMAS follow-up assessment, men aged 40 years and over who were Black and of Afro-Caribbean descent and South Asian men who were of Pakistani, Bangladeshi or Indian descent were invited to attend for the same suite of assessments. Ethnicity was defined by participants' self-report with 3 of 4 grandparents being of identical ethnic origin. Recruitment for these ethnic groups was through a combination of approaches including advertising in community centers and through local media targeted at the relevant ethnic groups. At their clinic visit, participants completed an interviewer-assisted questionnaire which included questions to determine their Physical Activity in the Elderly (PASE) score [11]. Smoking status was assessed by asking whether participants had ever smoked at least 100 cigarettes or been a regular pipe or cigar smoker. Those answering yes to any of the questions were considered as ever smokers. Ethical approval for the study was obtained in accordance with the local ethics review board in Manchester. All participants provided written informed consent.

### 2.2 Anthropometry

Height was measured to the nearest 1 mm using a stadiometer (Leicester Height Measure, SECA UK Ltd) and body weight was measured to the nearest 0.1 kg using an electronic scale (SECA UK Ltd). Body mass index (BMI) was calculated as weight in kilograms (kg) divided by the square of height (m).

### 2.3 Dual-energy X-ray absorptiometry

Participants had dual-energy X-ray absorptiometry (DXA) scans performed on QDR 4500A Discovery scanner, software version Apex 4.1 (Hologic Inc., Bedford, MA, USA). Measurements of aBMD at the whole body, total hip, femoral neck and lumbar spine (L1–4) were obtained; the non-dominant proximal femur was scanned. Hip axis length (HAL), whole body fat mass and lean mass were also measured. All scans were reported by an experienced musculoskeletal radiologist (JEA). Standard manufacturer QA and QC procedures were followed using manufacturer (Hologic) provided phantoms. The short term precision (co-efficient of variation (CV%)) in our center for repeat lumbar spine and proximal femur scans in adults (*n* = 22) was 1.1% and 1.3% respectively.

### 2.4 pQCT

Peripheral QCT (pQCT) measurements were made at the radius and tibia using a Stratec XCT-2000 scanner, software version 6.20 (Stratec, Pforzheim, Germany). All measurements were made in the non-dominant limb. Measurements were taken at 4%, 50% (radius) and 4%, 38%, (tibia) of the limb length which were measured using a wooden ruler (forearm) and segmometer (tibia). Forearm length was defined as the distance from the styloid process of the ulna to the olecranon. Leg length was defined as the distance from the most proximal edge of the medial malleolus to the intercondylar eminence. The scan sites were determined using a planar scout view of the distal radius or tibia and the reference line was placed to bisect the lateral border of the endplate. Total and trabecular vBMD (mg/mm^3^) and bone cross sectional area (CSA) (mm^2^) were measured at the 4% site (metaphysis). At the 50% radius and 38% tibia (diaphysis): CSA (mm^2^), cortical area (mm^2^), cortical vBMD (mg/mm^3^) were measured and cortical thickness (mm), CSMI (mm^4^) and SSI (mm^3^) were derived using the standard manufacturer protocol. Medullary area (mm^2^), was calculated by total area minus cortical area. CSMI and SSI are measures of bending and torsional strength at the diaphysis and have been related to fracture load [8], [9]. The software uses three image processing ‘modes’: contour mode determines the outer edges of the bone; peel mode is the method of separating cortical and sub-cortical bone from trabecular bone at metaphyseal sites and separation mode analyzes cortical bone at the diaphyseal sites. All scans were analyzed using contour mode 2 (manufacturer defined automated threshold of 169 mg/cm^3^), peel mode 1 which peels off the outer area of 55% of bone to leave the inner 45% area region of interest containing trabecular bone and marrow only. At the diaphysis, separation mode 1; threshold = 710 mg/cm^3^ was used for cortical vBMD and geometry and threshold = 480 mg/cm^3^ for SSI. All scans were reported by an experienced musculoskeletal radiologist (JEA). Where significant motion artefact was detected, scans were excluded. The short term precision of two repeat radius measurements with repositioning in adults were (*n* = 22): trabecular BMD 1.27%, 1.42%; cortical BMD 0.77%, 0.71%; cortical area 2.4%, 1.3%. Manufacturer's standard QA and QC procedures were followed using manufacturer supplied phantoms.

### 2.5 Statistical analysis

Descriptive statistics are presented as mean ± standard deviation (SD). Differences in descriptive characteristics were assessed using one-way analysis of variance with a Bonferroni multiple-comparison test. Differences in smoking status were assessed using a chi squared test. To investigate the ethnic differences in DXA and pQCT parameters, we performed linear regression analyses, with bone parameters as the dependent variable and ethnicity as the independent variable, with adjustments made for age, weight and height. We used the fitted regression lines to perform pairwise comparisons between ethnic groups, correcting for multiple comparisons using the Bonferroni method. All analyses were performed in Stata, Version 14.0 (StataCorp, College Station, TX, USA), and we considered results statistically significant at *p* < 0.05.

## 3 Results

### 3.1 Subject characteristics

Three hundred and forty three participants were included in the analyses, 235 White, 44 Black and 64 South Asian men. White men were older than the Black and South Asian men. South Asian men were shorter than White men (169.0 ± 5.8 vs 173.9 ± 6.9; < 0.001), weight and BMI were not significantly different between the three groups (Table 1); although South Asian men were lighter. Compared to Black and White men, total lean mass was lower in South Asian men, with no significant difference in total fat mass between groups. Whole body fat mass to lean mass ratio was higher in South Asian men compared to White and Black men. Hip axis length was shorter in Black and South Asian men compared to White men (Table 1). The percentage of subjects who were ever smokers was lower in South Asian compared to White men. Based on the PASE score, South Asian men had lower levels of physical activity than either Black or White men.

### 3.2 Differences in bone outcomes between Black and White men

Black men had higher aBMD at the whole body, total hip and femoral neck than White men. These differences persisted after adjustment for age, weight and height (Table 2). There were no significant differences in cortical or trabecular vBMD at the radius following adjustments (Table 3). At the diaphysis of the radius, Black men had thicker cortices and greater cortical area. As a consequence CSMI was 14% and SSI was 20% greater in Black men. At the diaphysis of the tibia, Black men had larger CSA and cortical area together with higher cortical vBMD, with 21% higher CSMI yet 29% lower SSI (Table 4). At the radius sites, Black men had larger bones than White men, though differences did not reach significance (*p* = 0.09 and *p* = 0.05 at the metaphysis and diaphysis respectively).

### 3.3 Differences in bone outcomes between White and South Asian men

There were no significant differences in aBMD between White and South Asians except at the whole body; this difference was attenuated and became nonsignificant after adjustment for age, height and weight (Table 2). South Asians had smaller CSA at the metaphysis and diaphysis of the radius, following adjustment, differences at the diaphysis were attenuated (Table 3). South Asians had smaller cortical area and consequently thinner cortices at the diaphysis of the radius and tibia, however, CSA and CSMI were similar to White men at both sites following adjustments; SSI was lower in South Asian men at the tibia yet similar at the radius compared to White men (Table 3, Table 4). For pQCT measured vBMD, South Asians had significantly lower trabecular vBMD at the metaphysis and higher cortical vBMD at the diaphysis of the tibia, both before and after adjustment compared to White men.

### 3.4 Differences in bone outcomes between Black and South Asian men

South Asian men had lower aBMD at the whole body, total hip, femoral neck and lumbar spine; the difference at the lumbar spine was attenuated following adjustments (Table 2). South Asians had smaller bones at the metaphysis and diaphysis of the radius by 12% and 8% respectively; after adjustment only differences at the metaphysis remained (Table 3). Thinner cortices and smaller cortical area all contributed to a 19% lower CSMI and 16% lower SSI at the diaphysis of the radius in South Asians. At the tibia, there were no significant differences in trabecular or cortical vBMD, however, South Asians had smaller bones, thinner cortices and lower cortical area with a 20% lower CSMI than Black men (Table 4).

## 4 Discussion

In this study, for the first time, we describe the ethnic differences in BMD, bone geometry and bone bending and torsional strength in UK men. Black men had higher aBMD compared to White and South Asian men, and these differences were independent of weight and height, in contrast differences in aBMD between White and South Asian men were attenuated by correcting for body size. We used pQCT to further understand the differences observed in aBMD measured by DXA. With the exception of cortical vBMD which was lower in White men compared to both Black and South Asian men, the differences in vBMD were far fewer than in DXA outcomes where Black men did not differ to White or South Asian men. Rather, the geometry of bone differed between the groups and mostly at the diaphyseal sites, and hip axis length was longer in White men. At the radius and tibia diaphysis, Black men had more cortical bone within a slightly larger periosteal envelope and consequently greater bending strength than the other two groups. For the same size and body weight, South Asian men had similar sized bones compared to White and Black men at the diaphysis but had thinner cortices. Patterns were similar in the radius and tibia. At the metaphysis, South Asian men had smaller bones but similar vBMD to White men. Taken together these observations suggest that there are other factors than aBMD which contribute to the differences in fracture risk between the two groups [1].

In this study we show that Black men had higher aBMD compared to White and South Asian men – independent of differences in body size. Consistent with our findings, total hip aBMD was shown to be higher in Black Afro-Caribbean men compared to White American men [12]. The same group extended these findings by showing that the highest prevalence of fracture was in White American men and the lowest was observed in Black Afro-Caribbean men [13]. Larger bones containing greater cortical area have been associated with greater bone bending strength as estimated by CSMI [14]. Our data are consistent with previous findings with Black men having larger bones, thicker cortices and greater CSMI compared to White and South Asian men. Additionally White men had longer hip axis lengths than both Black and South Asian men, which has also been shown to be a risk factor for fracture [15]. Collectively, these data suggest an ‘advantageous’ phenotype with greater strength in bending and torsion in Black men, potentially decreasing the risk of fracture [7], [8].

Our findings that after adjustment for covariates, there were no differences in aBMD between White and South Asian men is consistent with data in young women were observed differences in hip and lumbar spine aBMD between White and South Asian women were explained by body size [3]. Furthermore, The Oslo Health Study showed no differences in distal or ultra-distal forearm aBMD between South Asian and White Norwegian men and women independent of height adjustments, though interestingly, bone mineral apparent density (BMAD) was shown to be greater in South Asian than in Norwegian men and women [16]. Together these studies show that there are no differences in aBMD between South Asian men and women when compared to White Europeans. In contrast to these findings comparing aBMD differences, we report differences in bone geometry in South Asian compared to White men. South Asian men had smaller CSA at the tibia compared to White men, these differences were attenuated after adjustments were made for age, weight and height. Despite similar CSA at the diaphysis, South Asian men had thinner cortices but higher cortical vBMD and similar strength. Lower cortical vBMD at the diaphysis of the tibia in White men may indicate increased cortical porosity and bone turnover in this group which may in part contribute to higher fracture risk; greater porosity may be related to the differences in age between the groups but the difference was robust to age-adjustments [1]. Our findings are similar to those reported in young British women, where South Asians had significantly thinner cortices but similar bone size and strength at the diaphysis when compared to White European women [6]. Parallel to data in young women, a recent study in older UK women showed that following adjustments for BMI, South Asians had smaller bones and cortical area, with lower bone mineral content at the diaphyseal radius compared to White European women [17]. Further, a Finnish study showed that South Asian women had lower cortical vBMD and area at the diaphyseal radius, and smaller bones when compared to White Finnish women [18].

Surrogates of bone fracture load (CSMI and SSI) were used to describe bone strength at the diaphysis. These have been shown to predict fracture load in laboratory testing [8]. The findings of lower SSI in Black men were surprising. These measures are derived parameters and are heavily based on cross-sectional area which is only one component of bone strength. It is important to note that SSI also includes a measure of vBMD [7]. We expected that SSI at the tibia would be greater in Black than in White men given the higher vBMD and greater CSA, the parameters from which SSI is derived. The most plausible explanation for this finding is that the measurement of SSI is not fully robust to differing bone shape, distribution and density and therefore this parameter may be limited in comparisons between populations where bone shape may differ.

There are several potential limitations in this study. Ancestral origins were self-reported and it is possible that this may have resulted in misclassification; we attempted to limit this however, by ensuring that 3 out of 4 grandparents were of the same ethnic origin. The concept of ethnicity embraces also cultural and environmental differences, however, this was beyond the scope of this study. The focus in this study was on looking at ethnic differences in bone parameters. There was some evidence that levels of physical activity and smoking varied between the groups of men which may have contributed to the observed differences. The number of men in the Black and South Asian groups was relatively small and so caution is required in interpreting the results. Further adequately powered studies are needed to explore in more detail our findings and the role of lifestyle and other factors which contribute to observed ethnic differences in bone health in these groups. It should also be stated that this is a cross-sectional study so we were unable to study antecedents of the observed ethnic differences in the bone outcomes. We do not have data on fracture so we cannot draw causal associations between bone health and fracture.

In conclusion, this study demonstrated that Black men have higher aBMD compared to White and South Asian men, with no differences between the latter two groups. Greater aBMD is likely due to the Black men having wider bones with thicker cortices, as indicated by the pQCT data. These differences are reflected in the greater bone strength in Black men. South Asian men had thinner cortices at the radius and tibia, however, bone strength appeared to be maintained as there were no differences in CSMI when compared to White men. These data indicate the necessity to understand the underlying ethnic differences in bone shape, mineralization and distribution to ultimately decrease the burden of male osteoporosis.

## Figures and Tables

**Table 1:** Descriptive characteristics.

	Mean ± SD			Group differences (*p*-values)		
	White *(n* *=* *235)*	Black *(n* *=* *44)*	South Asian *(n* *=* *64)*	White vs Black	White vs South Asian	South Asian vs Black
Age (yr)	64.7 ± 10.7	59.7 ± 11.3	59.2 ± 10.9	**0.016**	**0.001**	1.00
Height (cm)	173.9 ± 6.9	171.9 ± 7.2	169.0 ± 5.8	0.24	**< 0.001**	0.09
Weight (kg)	84.4 ± 12.4	85.3 ± 12.8	80.7 ± 11.3	1.00	0.10	0.17
BMI (kg/m^2^)	27.9 ± 3.5	28.8 ± 4.0	28.2 ± 3.4	0.31	1.00	1.00
Whole body fat mass (kg)^a^	24.7 ± 6.6	22.8 ± 6.3	25.9 ± 6.7	0.25	0.69	0.06
Whole body lean mass (kg)^a^	56.8 ± 7.4	58.7 ± 7.1	52.3 ± 6.3	0.32	**< 0.001**	**< 0.001**
Whole body fat:lean mass ratio^a^	0.43 ± 0.1	0.39 ± 0.1	0.49 ± 0.1	**0.014**	**< 0.0001**	**< 0.0001**
Hip axis length (mm)^a^	121.9 ± 6.9	117.5 ± 8.3	116.0 ± 7.3	**0.002**	**< 0.001**	1.00
PASE score	201 ± 91	231 ± 96	159 ± 101	0.31	**0.009**	**0.003**
Ever smoked (n, %)	127 (55)	18 (46)	23 (38)	0.29	**0.015**	0.40

All values are mean ± SD. ^a^indicates measures are from DXA; PASE: Physical Activity Scale for the Elderly; bold indicates *p* < 0.05.

**Table 2:** Differences in DXA outcomes between ethnic groups.

	Mean ± SD			Group differences (*p*-values)		
	White *(n* *=* *235)*	Black *(n* *=* *44)*	South Asian *(n* *=* *64)*	White vs Black	White vs South Asian	South Asian vs Black
*Whole body aBMD (g/cm^2^)*						
Unadjusted	1.16 ± 0.11	1.23 ± 0.13	1.11 ± 0.08	**0.001**	**0.002**	**< 0.0001**
Adjusted	1.15 ± 0.11	1.23 ± 0.11	1.12 ± 0.11	**< 0.0001**	0.09	**< 0.0001**

*Total Hip aBMD (g/cm^2^)*						
Unadjusted	1.02 ± 0.15	1.15 ± 0.17	1.03 ± 0.13	**< 0.0001**	1.00	**< 0.0001**
Adjusted	1.02 ± 0.14	1.15 ± 0.13	1.05 ± 0.14	**< 0.0001**	0.41	**0.001**

*Femoral neck aBMD (g/cm^2^)*						
Unadjusted	0.82 ± 0.13	0.93 ± 0.16	0.84 ± 0.12	**< 0.0001**	0.78	**0.002**
Adjusted	0.81 ± 0.13	0.93 ± 0.13	0.85 ± 0.13	**< 0.0001**	0.10	**0.01**

*Lumbar spine aBMD (g/cm^2^)*						
Unadjusted	1.10 ± 0.22	1.14 ± 0.18	1.04 ± 0.15	0.84	0.10	**0.04**
Adjusted	1.09 ± 0.20	1.15 ± 0.19	1.07 ± 0.20	0.26	1.00	0.19

All values are mean ± SD. Adjusted mean ± SD from a linear regression model with adjustments for age (yr), weight (kg) and height (cm), bold indicates *p* < 0.05.

**Table 3:** Ethnic differences at the radius using pQCT parameters.

	Mean ± SD			Group differences (*p*-values)		
	White *(n* *=* *242)*	Black *(n* *=* *42)*	South Asian *(n* *=* *64)*	White vs Black	White vs South Asian	South Asian vs Black
*4% Radius*						
Total vBMD (mg/cm^3^)						
Unadjusted	434.0 ± 70.2	447.1 ± 93.0	453.5 ± 70.2	0.83	0.18	1.00
Adjusted	436.8 ± 73.3	440.5 ± 72.4	447.3 ± 75.3	1.00	1.00	1.00
Trabecular vBMD (mg/cm^3^)						
Unadjusted	207.1 ± 47.0	201.3 ± 65.1	196.6 ± 48.4	1.00	0.46	1.00
Adjusted	207.4 ± 51.1	199.6 ± 50.4	194.8 ± 52.4	1.00	0.28	1.00
CSA (mm^2^)						
Unadjusted	346.5 ± 52.8	356.4 ± 49.8	304.9 ± 41.3	0.70	**< 0.0001**	**< 0.0001**
Adjusted	342.6 ± 48.2	359.7 ± 47.6	317.4 ± 49.5	0.09	**0.001**	**< 0.0001**

*50% Radius*						
Ct. vBMD (mg/cm^3^)						
Unadjusted	1211.6 ± 30.6	1224.4 ± 29.2	1216.6 ± 30.6	**0.03**	0.71	0.59
Adjusted	1212.1 ± 30.6	1222.8 ± 30.2	1215.6 ± 31.4	0.11	1.00	0.67
Ct. Thickness (mm)						
Unadjusted	3.2 ± 0.4	3.7 ± 0.4	3.0 ± 0.4	**< 0.0001**	**< 0.0001**	**< 0.0001**
Adjusted	3.2 ± 0.4	3.7 ± 0.4	3.0 ± 0.4	**< 0.0001**	**0.001**	**< 0.0001**
CSA (mm^2^)						
Unadjusted	151.2 ± 22.3	156.0 ± 17.9	140.0 ± 18.3	0.52	**0.001**	**< 0.0001**
Adjusted	149.5 ± 20.4	157.7 ± 20.2	145.0 ± 21.0	0.05	0.39	**0.005**
Ct. Area (mm^2^)						
Unadjusted	106.9 ± 14.4	119.2 ± 11.5	96.0 ± 11.4	**< 0.0001**	**< 0.0001**	**< 0.0001**
Adjusted	106.1 ± 12.6	119.5 ± 12.4	98.8 ± 12.9	**< 0.0001**	**< 0.0001**	**< 0.0001**
Medullary Area (mm^2^)						
Unadjusted	44.3 ± 17.3	36.7 ± 13.1	44.0 ± 14.1	**0.02**	1.00	0.07
Adjusted	43.5 ± 16.4	38.1 ± 16.2	46.2 ± 16.8	0.15	0.80	**0.04**
CSMI (mm^4^)						
Unadjusted	1678.7 ± 454.9	1840.0 ± 387.3	1416.7 ± 329.6	0.70	**< 0.0001**	**< 0.0001**
Adjusted	1645.3 ± 406.5	1870.6 ± 401.2	1520.8 ± 417.0	**0.003**	0.12	**< 0.0001**
SSI (mm^3^)						
Unadjusted	335.8 ± 62.6	396.1 ± 58.9	321.1 ± 54.0	**< 0.0001**	0.26	**< 0.0001**
Adjusted	331.8 ± 57.9	398.8 ± 57.1	334.1 ± 59.4	**< 0.0001**	1.00	**< 0.0001**

All values are mean ± SD. Adjusted mean ± SD from a linear regression model with adjustments for age (yr), weight (kg) and height (cm), bold indicates *p* < 0.05. vBMD, volumetric bone mineral density; CSA, cross-sectional area; Ct, cortical; CSMI, cross-sectional moment of inertia; SSI, stress strain index.

**Table 4:** Ethnic differences at the tibia using pQCT parameters.

	Mean ± SD			Group differences (*p*-values)		
	White *(n* *=* *216)*	Black *(n* *=* *42)*	South Asian *(n* *=* *61)*	White vs Black	White vs South Asian	South Asian vs Black
*4% Tibia*						
Total vBMD (mg/cm^3^)						
Unadjusted	321.2 ± 43.0	334.4 ± 62.7	315.4 ± 42.2	0.27	1.00	0.13
Adjusted	322.8 ± 45.8	331.0 ± 45.1	312.2 ± 46.8	0.86	0.40	0.12
Trabecular vBMD (mg/cm^3^)						
Unadjusted	243.8 ± 36.6	238.7 ± 49.2	226.5 ± 31.8	1.00	**0.006**	0.33
Adjusted	244.6 ± 38.2	237.1 ± 37.7	224.9 ± 39.1	0.73	**0.003**	0.33
CSA (mm^2^)						
Unadjusted	1269.7 ± 168.9	1198.5 ± 161.4	1140.9 ± 128.3	**0.03**	**< 0.0001**	0.23
Adjusted	1251.6 ± 143.9	1217.9 ± 141.9	1192.4 ± 147.2	0.50	**0.02**	1.00

*38% Tibia*						
Ct. vBMD (mg/cm^3^)						
Unadjusted	1172.9 ± 39.0	1225.9 ± 20.2	1212.6 ± 26.2	**< 0.0001**	**< 0.0001**	0.18
Adjusted	1173.2 ± 35.4	1225.2 ± 34.9	1212.1 ± 36.2	**< 0.0001**	**< 0.0001**	0.18
Ct. Thickness (mm)						
Unadjusted	5.9 ± 0.8	6.2 ± 0.7	5.5 ± 0.6	**0.03**	**0.007**	**< 0.0001**
Adjusted	5.9 ± 0.7	6.1 ± 0.7	5.5 ± 0.7	0.11	**0.002**	**< 0.0001**
CSA (mm^2^)						
Unadjusted	477.1 ± 53.1	515.2 ± 58.4	452.9 ± 46.5	**< 0.0001**	**0.008**	**< 0.0001**
Adjusted	472.4 ± 46.2	519.4 ± 45.6	469.4 ± 47.3	**< 0.0001**	1.00	**< 0.0001**
Ct. Area (mm^2^)						
Unadjusted	344.8 ± 44.0	376.5 ± 45.1	321.1 ± 36.6	**< 0.0001**	**0.001**	**< 0.0001**
Adjusted	343.3 ± 39.8	376.0 ± 39.2	326.7 ± 40.7	**< 0.0001**	**0.019**	**< 0.0001**
Medullary Area (mm^2^)						
Unadjusted	132.7 ± 39.8	138.7 ± 39.2	133.1 ± 32.6	1.00	1.00	1.00
Adjusted	129.2 ± 37.0	143.3 ± 36.5	142.5 ± 37.9	0.07	0.05	1.00
CSMI (mm^4^)						
Unadjusted	16,843.3 ± 3625.3	19,735.4 ± 4306.8	15,102.9 ± 3020.3	**< 0.0001**	**0.003**	**< 0.0001**
Adjusted	16,547.9 ± 3187.6	19,936.8 ± 3143.1	16,010.1 ± 3259.2	**< 0.0001**	0.80	**< 0.0001**
SSI (mm^3^)						
Unadjusted	1628.2 ± 466.3	1155.8 ± 386.0	1140.3 ± 386.0	**< 0.0001**	**< 0.0001**	1.00
Adjusted	1622.7 ± 394.1	1158.5 ± 388.6	1157.7 ± 403.0	**< 0.0001**	**< 0.0001**	1.00

All values are mean ± SD. Adjusted mean ± SD from a linear regression model with adjustments for age (yr), weight (kg) and height (cm), bold indicates *p* < 0.05. vBMD, volumetric bone mineral density; CSA, cross-sectional area; Ct, cortical; CSMI, cross-sectional moment of inertia; SSI, stress strain index.

## References

[1] Curtis E.M., van der Velde R., Moon R.J., van den Bergh J.P.W., Geusens P., de Vries F., van Staa T.P., Cooper C., Harvey N.C. (2016). Epidemiology of fractures in the United Kingdom 1988–2012: variation with age, sex, geography, ethnicity and socioeconomic status. Bone.

[2] Tobias J.H., Cook D.G., Chambers T.J., Dalzell N. (1994). A comparison of bone mineral density between Caucasian, Asian and Afro-Caribbean women. Clin. Sci. (Lond.).

[3] Roy D., Swarbrick C., King Y., Pye S., Adams J., Berry J., Silman A., O'Neill T. (2005). Differences in peak bone mass in women of European and South Asian origin can be explained by differences in body size. Osteoporos. Int..

[4] Ward K. (2012). Musculoskeletal phenotype through the life course: the role of nutrition. Proc. Nutr. Soc..

[5] Zengin A., Prentice A., Ward K.A. (2015). Ethnic differences in bone health. Front. Endocrinol..

[6] Ward K.A., Roy D.K., Pye S.R., O'Neill T.W., Berry J.L., Swarbrick C.M., Silman A.J., Adams J.E. (2007). Forearm bone geometry and mineral content in UK women of European and South-Asian origin. Bone.

[7] Capozza R.F., Feldman S., Mortarino P., Reina P.S., Schiessl H., Rittweger J., Ferretti J.L., Cointry G.R. (2010). Structural analysis of the human tibia by tomographic (pQCT) serial scans. J. Anat..

[8] Ferretti J.L., Capozza R.F., Zanchetta J.R. (1996). Mechanical validation of a tomographic (pQCT) index for noninvasive estimation of rat femur bending strength. Bone.

[9] Schiessl H., Feretti J.L., Tysarczyk-Niemeyer G., Willnecker J. (1996). Noninvasive bone strength index as analyzed by peripheral quantitative computed tomography (pQCT). Paediatric Osteology: New Developments in Diagnostics and Therapy.

[10] Lee D.M., Pye S.R., Tajar A., O'Neill T.W., Finn J.D., Boonen S., Bartfai G., Casanueva F.F., Forti G., Giwercman A., Han T.S., Huhtaniemi I.T., Kula K., Lean M.E., Pendleton N., Punab M., Silman A.J., Vanderschueren D., Wu F.C., group Es (2013). Cohort profile: the European male ageing study. Int. J. Epidemiol..

[11] Washburn R.A., Smith K.W., Jette A.M., Janney C.A. (1993). The physical activity scale for the elderly (PASE): development and evaluation. J. Clin. Epidemiol..

[12] Nam H.S., Shin M.H., Zmuda J.M., Leung P.C., Barrett-Connor E., Orwoll E.S., Cauley J.A. (2010). Race/ethnic differences in bone mineral densities in older men. Osteoporos. Int..

[13] Shin M.H., Zmuda J.M., Barrett-Connor E., Sheu Y., Patrick A.L., Leung P.C., Kwok A., Kweon S.S., Nam H.S., Cauley J.A. (2014). Race/ethnic differences in associations between bone mineral density and fracture history in older men. Osteoporos. Int..

[14] Hamilton C.J., Thomas S.G., Jamal S.A. (2010). Associations between leisure physical activity participation and cortical bone mass and geometry at the radius and tibia in a Canadian cohort of postmenopausal women. Bone.

[15] Crabtree N.J., Kroger H., Martin A., Pols H.A., Lorenc R., Nijs J., Stepan J.J., Falch J.A., Miazgowski T., Grazio S., Raptou P., Adams J., Collings A., Khaw K.T., Rushton N., Lunt M., Dixon A.K., Reeve J. (2002). Improving risk assessment: Hip geometry, bone mineral distribution and bone strength in hip fracture cases and controls. The EPOS study. European prospective osteoporosis study. Osteoporos. Int..

[16] Alver K., Meyer H.E., Falch J.A., Sogaard A.J. (2005). Bone mineral density in ethnic Norwegians and Pakistani immigrants living in Oslo—the Oslo health study. Osteoporos. Int..

[17] Darling A.L., Hakim O.A., Horton K., Gibbs M.A., Cui L., Berry J.L., Lanham-New S.A., Hart K.H. (2013). Adaptations in tibial cortical thickness and total volumetric bone density in postmenopausal South Asian women with small bone size. Bone.

[18] Islam M.Z., Viljakainen H.T., Karkkainen M.U., Saarnio E., Laitinen K., Lamberg-Allardt C. (2012). Prevalence of vitamin D deficiency and secondary hyperparathyroidism during winter in pre-menopausal Bangladeshi and Somali immigrant and ethnic Finnish women: associations with forearm bone mineral density. Br. J. Nutr..

